# Ultrasensitive and Adjustable Nanothermometers Based on Er^3+^-Sensitized Core@Shell Nanoparticles for Use in the First Biological Window

**DOI:** 10.1021/acsami.4c10176

**Published:** 2024-10-04

**Authors:** Tomasz Grzyb, Sylwia Ryszczyńska, Natalia Jurga, Dominika Przybylska, Inocencio R. Martín

**Affiliations:** † Department of Rare Earths, Faculty of Chemistry, Adam Mickiewicz University in Poznań, Uniwersytetu Poznańskiego 8, Poznań 61-614, Poland; ‡ Department of Chemistry, Faculty of Forestry and Wood Technology, Poznan University of Life Sciences, Wojska Polskiego 75, Poznań 60-625, Poland; § Departamento de Fisica, Instituto de Materiales y Nanotecnología (IMN), Universidad de La Laguna, San Cristóbal de La Laguna 38200, Santa Cruz de Tenerife, Spain

**Keywords:** luminescence, upconversion, nanoparticles, lanthanide ions, temperature sensors, biological windows

## Abstract

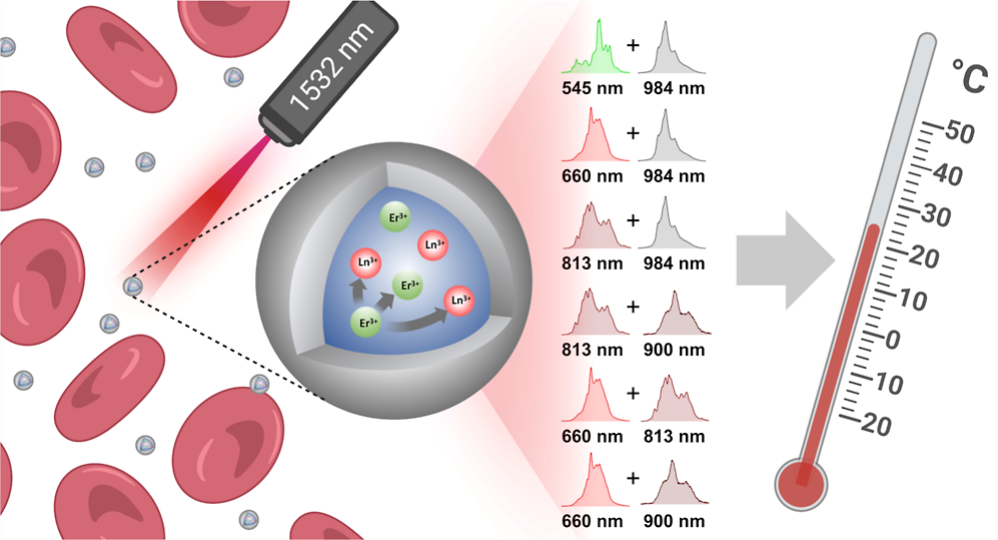

In recent years, intensive research has focused on lanthanide-doped nanoparticles (NPs) used as noncontact temperature sensors, particularly in nanomedicine. These NPs must be capable of excitation and emission within biological windows, where biological materials usually show better transparency for radiation. In this article, we propose that NPs sensitized with Er^3+^ ions can be applied as temperature sensors in biological materials. We synthesized the NPs through a reaction in high-boiling solvents and confirmed their crystal structure and the formation of core@shell NPs by using X-ray diffraction, high-resolution transmission electron microscopy, and element distribution mapping within the NPs. NaErF_4_@NaYF_4_, NaYF_4_:12.5% Er^3+^, 2.5% Tm^3+^@NaYF_4_, NaYF_4_:7.5% Er^3+^@NaYF_4_, and NaYF_4_:12.5% Er^3+^, 2.5% Ho^3+^@NaYF_4_ exhibited intense upconversion (UC) emission under 1532 nm laser excitation detectable also in the whole human blood. We propose that this UC results from energy transfer between Er^3+^ ions and from Er^3+^ to Tm^3+^ or Ho^3+^ codopants. To determine the mechanism of UC, we measured the dependence of the emission band intensities on the laser power densities. Importantly, we also analyzed the temperature-dependent emission of the NPs within the 295–360 K range. Based on the collected emission spectra, we calculated the luminescence intensity ratios (LIRs) of the emission bands to assess their potential for optical temperature sensing. The temperature-sensing properties varied with the concentration of Er^3+^ ions and the presence of additional Tm^3+^ or Ho^3+^ codopants. Depending on the NP composition and the emission bands used for luminescence ratio calculations, the maximum relative temperature sensitivity ranged from 4.55%·K^–1^ to 1.12%·K^–1^, with temperature resolution between 0.05 and 2.53 K at room temperature. Finally, as proof of using NPs as temperature sensors in biomedicine, we successfully measured the temperature-dependent emission of NaYF_4_:7.5% Er^3+^@NaYF_4_ NPs dispersed in whole blood under 1532 nm excitation. We demonstrated that the ratio of Er^3+^ ion emission bands changes with temperature, indicating that these NPs have potential applications in temperature sensing within biological environments. We also confirmed the properties of NPs as temperature sensors by measuring the temperature reading uncertainty and the repeatability of the LIR readings during heating–cooling cycles, thereby confirming the excellent properties of the studied systems as temperature sensors.

## 1 Introduction

Luminescent nanoparticles (NPs) containing lanthanide ions (Ln^3+^) have been intensively investigated in the last years as they offer unique properties and are demanded in analytical applications,^1−3^ anticounterfeiting,^4,5^ forensic sciences,^6,7^ nanomedicine for bioimaging, drug delivery, photothermal therapy, as contrast agents, and in cancer treatment.^8−12^ One of the most developed fields in which Ln^3+^-doped NPs are researched and advanced is that of various types of sensors, such as temperature or pressure.^13−17^ These applications result from the spectroscopic properties of Ln^3+^ ions, precisely the nature of the f–f electronic transitions responsible for their emission.^18,19^ The luminescent bands of Ln^3+^ ions have maxima at specific wavelengths characteristic of each ion. These bands are typically narrow because f-levels are shielded by electrons from the higher subshells. Consequently, changes in the crystal field do not significantly affect the electronic states of Ln^3+^ ions, unlike the case with d-electronic elements. Moreover, Ln^3+^-doped NPs can emit radiation over a wide range, from ultraviolet (UV) to near-infrared (NIR).^20,21^

In the case of using Ln^3+^-doped NPs as sensors, the most important aspect is that the ratio of individual emission bands can change under the influence of an external factor such as ambient temperature. A particular case is when the emitting levels of the Ln^3+^ ions are thermally coupled, *e.g.*, ^2^H_11_ and ^4^S_3/2_ energy states of Er^3+^ ions, responsible for the green emission.^21,22^ This is significant because it allows for noncontact temperature measurement simply by observing the luminescence of the NPs, including the NIR range.^21^ Another feature of Ln^3+^-doped NPs is the ability to show the anti-Stokes emission *via* conversion of NIR radiation to shorter wavelengths, usually in the visible range, called upconversion (UC).^23,24^ This is particularly crucial when using NPs in nanomedicine, where the tissue or blood is transparent only to limited spectral ranges, known as biological windows (BWs).^25,26^ There are three spectral ranges, called first BW (≈650–950 nm), second BW (≈1000–1350 nm), and third BW (≈1500–1800 nm), in which the Ln^3+^-doped NPs can be excited and luminescence detected.^25,27−29^ Therefore, one of the intensively developing areas, which is nanothermometry for biological and medical applications, involves the designing and study of NPs that enable efficient radiation conversion within BWs and have high sensitivity to changes in temperature.^30^

Within the range of the third BW, a few Ln^3+^ ions possess absorption bands. These include Sm^3+^ (one band has a maximum of around 1500 nm), Er^3+^ (1530 nm), Pr^3+^ (1550 nm), Tm^3+^ (1630 nm), and Dy^3+^ (1700 nm).^31^ However, from the ions mentioned above, only Er^3+^ ions can be excited, resulting in UC luminescence or energy transfer (ET) to different ions, precisely under 1500–1550 nm laser radiation.^32,33^

Upconverting NPs doped with Er^3+^ are the subject of many studies, mostly when the NPs are codoped with Yb^3+^ ions, which allow for their excitation at around 975 nm.^34−38^ However, because of their electronic structure, Er^3+^ ions can undergo UC phenomena under 975 nm without Yb^3+^ ions, thanks to the ^4^I_15/2_ → ^4^I_11/2_ ground-state absorption (GSA) followed by the excited-state absorption (ESA) process.^39,40^ Absorption spectra of materials containing Er^3+^ ions also prove the possibility of their excitation at around 808 and 1500 nm *via*^4^I_15/2_ → ^4^I_9/2_ or ^4^I_15/2_ → ^4^I_13/2_ transitions, respectively.^41^ The absorption band related to the ^4^I_15/2_ → ^4^I_13/2_ transition is intense and broad, covering a range from 1450 to 1550 nm. That fact is promising for using this range for excitation and generating UC luminescence as it lies within the third BW, in contradiction to the 975 nm excitation wavelength, which is between the first and second BW and has limited applicability for biomedicine. Indeed, the UC under excitation in the mentioned range was the subject of several studies, revealing that the upconversion quantum yield (UCQY) of the emission under excitation at around 1500 nm is comparable with that for Yb^3+^/Er^3+^-doped NaYF_4_ NPs excited *via* 975 nm, *e.g.*, 1.2 ± 0.1% (1490 nm, 150 W·cm^–2^) measured for LiYF_4_:10% Er^3+^ NPs, 2.01 ± 0.19% (1523 nm, 0.43 W·cm^–2^) determined for NaYF_4_:28% Er^3+^@NaLuF_4_ NPs in PMMA, or 3.9 ± 0.3% in the case of NaYF_4_:10% Er^3+^@NaYF_4_ NPs (1523 nm, 18 W·cm^–2^).^42−44^ Typical upconverting Yb^3+^/Er^3+^-doped NPs considered as good-emitting show UCQY in the range of 0.2–9% under 975 nm excitation with similar power densities to those mentioned above, whereas 9% is currently the highest value published so far.^45−47^

Under irradiation with a laser line within the third BW, Er^3+^ ions undergo excitation *via* GSA and ESA processes, as well as self-sensitization, *i.e.*, ET between them, resulting in emission within the first BW at around 800 nm, thanks to the ^4^I_9/2_ → ^4^I_15/2_ electronic transition. However, emission with a maximum at 970 nm is also possible due to the ^4^I_11/2_ → ^4^I_15/2_ transition, which, although it falls outside the range of BWs, has a broad emission spectrum, and a portion of it falls within the first BW, making it usable in nanothermometry. For example, in our previous study, we demonstrated that the luminescence intensity ratio (LIR) of 800 and 970 nm emission bands in SrF_2_:Er^3+^ NPs observed under 1532 nm excitation can be temperature-sensitive, achieving a relative sensitivity of around 0.93%·K^–1^ at 373 K, comparable to other reports on upconverting NPs.^39^ In these studies, we have also examined how the relative sensitivity varies with different concentrations of Er^3+^.^39^ This is the only research that has explored this specific aspect.

In this article, we have expanded the scope to include NPs codoped with other Ln^3+^ ions, where ET is possible from Er^3+^ ions excited under 1532 nm. Introducing other Ln^3+^ emitters allows the observation of additional emission bands whose temperature-related behavior may differ from Er^3+^ ions. Furthermore, we have tested a proof-of-concept idea to analyze if changes in the ratio between emission lines of Ln^3+^ emitters under 1532 nm excitation can be detected in the challenging environment of whole blood. Our results indicate that the proposed types of NPs have great potential as nanothermometers for biomedical applications.

## 2 Materials and Methods

### 2.1 Materials

For the synthesis of rare earth chloride precursors, we used the following reagents: yttrium, holmium, erbium, and thulium oxides (Y_2_O_3_, Ho_2_O_3_, Er_2_O_3_, and Tm_2_O_3_, 99.99%, Alfa-Aesar, Germany) and hydrochloric acid (HCl, 37%, ultrapure, Sigma-Aldrich, Poland). Sodium oleate (≥82%, Sigma-Aldrich, Poland) and ammonium fluoride (NH_4_F, ≥98%, Alfa Aesar, Germany) were used as sources of Na^+^ and F^–^ ions, respectively. The reaction was performed in *n*-octadecene (OD, 90%, Alfa Aesar, Germany) and oleic acid (OA, extra pure, Fisher Scientific, Germany). Ethanol (98.8%, Avantor, Poland) and *n*-hexane (95%, VWR Chemicals, Poland) were used to purify the prepared NPs.

### 2.2 Synthesis of Rare Earth Chlorides

Yttrium, holmium, erbium, and thulium oxides were dissolved separately in an aqueous solution of hydrochloric acid (6 mL of 2 M HCl per 1 mmol of RE oxide) at 70 °C and stirred overnight in a reflux condenser. A clear solution was evaporated under a vacuum by using a rotary evaporator. The RE chlorides were dried next at 110 °C overnight under atmospheric pressure.

### 2.3 Synthesis of Core@Shell NPs

To synthesize core and core@shell NPs, we used the previously reported precipitation reaction of NaREF_4_ fluorides in high-temperature boiling point solvents, *i.e.*, OA and OD and from the RECl_3_ salts in the presence of NH_4_F. The synthesis of core@shell NPs was divided into three steps: (I) synthesis of β-core NPs (with a hexagonal structure), (II) synthesis of α-shell precursor NPs (with cubic structure), and (III) synthesis of β-core@β-shell NPs.^48,49^

#### 2.3.1 β-Core-NaYF_4_:Ln^3+^ NPs

To obtain 5 mmol of β-NaYF_4_:Ln^3+^ NPs (where Ln = Ho, Er, or Tm), 5 mmol of RECl_3_ chlorides was placed in a three-neck round-bottom flask, mixed in a stoichiometric ratio depending on the Ln^3+^ concentration; *e.g*., to obtain β-NaYF_4_:12.5% Er^3+^, 2.5% Ho^3+^ NPs, 4.25 mmol of YCl_3_, 0.625 mmol of ErCl_3_, and 0.125 mmol of HoCl_3_ were used. Next, 100 mL of OD and OA mixture (1:1) was mixed with chlorides, and the reagents were heated to 100 °C under vacuum for 150 min and stirred to remove water from the mixture. In the next step, 10 mmol of sodium oleate and 30 mmol of NH_4_F were added to the heated mixture in a nitrogen atmosphere and then degassed again under vacuum at 100 °C for 30 min. After that, the mixture was heated to 300 °C under vigorous stirring and nitrogen flow for 60 min and then cooled down. The postreaction product was centrifuged (10 min, 9000 rpm) and purified five times by sequential dispersing in *n*-hexane and precipitating the NPs with ethanol (5 min, 8000 rpm). The obtained NPs were redispersed in *n*-hexane for water-soluble colloids preparation or air-dried for analyses requiring powder form.

#### 2.3.2 α-Shell-Precursor NaYF_4_ NPs

To obtain 15 mmol of α-NaYF_4_ NPs, 300 mL of OD and OA mixture (1:1) and 15 mmol of YCl_3_ were purified at 100 °C under vacuum for 180 min. Next, 22.5 mmol of sodium oleate and 60.0 mmol of NH_4_F were added to the heated mixture under a nitrogen flow and outgassed at 100 °C under vacuum for 45 min. The mixture was heated at 200 °C with vigorous stirring, under nitrogen flow for 60 min, and cooled down. The postreaction mixture was centrifuged (10 min, 9000 rpm), and the product was precipitated by adding ethanol and purified three times by sequential dispersion in *n*-hexane and precipitation with ethanol (5 min, 8000 rpm). The obtained NPs were air-dried.

#### 2.3.3 β-Core@β-Shell NPs

To obtain β-NaYF_4_:Ln^3+^@β-NaYF_4_ NPs, 1 mmol of β-core-NaYF_4_:Ln^3+^ NPs and 7 mmol of α-NaYF_4_ NPs were added to 32 mL of OD and OA mixture (1:1) and degassed at 100 °C under vacuum for 180 min. Then, the mixture was heated at 300 °C with vigorous stirring, under nitrogen flow for 135 min, and cooled down. The postreaction mixture was centrifuged (10 min, 9000 rpm) and purified four times by sequential dispersion in *n*-hexane and precipitation with ethanol (5 min, 8000 rpm). The obtained NPs were redispersed in *n*-hexane (100 mg of NPs in 5 mL of hexane) or air-dried for analyses requiring powder form. In the remaining part of the article, we use the simplified notation core@shell NPs to refer to β-core@β-shell NPs.

### 2.4 Synthesis of Ligand-Free NaErF_4_@NaYF_4_ and NaYF_4_:7.5% Er^3+^@NaYF_4_ Nanoparticles

Changing the properties of NPs from hydrophobic to hydrophilic is needed for biomedical applications. The NPs’ surface modification has been conducted using our previously described procedure.^50^ 2.5 mL of 2 M HCl was added to the OA-capped NaErF_4_@NaYF_4_ (or NaYF_4_:7.5% Er^3+^@NaYF_4_) NPs in the form of their hexane colloid received directly after their synthesis to protonate and remove OA from the NP’s surface. The mixture was vigorously stirred for 15 min at room temperature. After that, the particles were successfully transferred to a water solution, as confirmed by dynamic light scattering (DLS) analysis (the absence of NPs in the organic phase). Next, the mixture was ultrasonicated for 5 min. The product was collected by centrifugation at 9000 rpm for 10 min and washed with a mixture of water and ethanol (in a 1:1 ratio) to discard the organic layer containing the OA molecules. The washing procedure was repeated two times. The ligand-free NPs were dispersed in distilled water and stored at 4 °C for further studies.

### 2.5 Characterization of Materials

The materials were characterized by using selected analytical techniques. TGA was carried out to analyze the water content in RECl_3_ and the organic phase in the obtained products using a thermogravimetric analyzer (TGA 4000,PerkinElmer), under nitrogen flow, in the temperature range of 30–600 °C with a temperature ramp of 10 °C·min^–1^.

X-ray diffraction (XRD) analysis of NPs in the form of powders was performed using a Bruker AXS D8 ADVANCE diffractometer equipment with Cu Kα radiation (λ = 0.154 nm), with a step size of 0.05° in the angle range of 2θ = 10–80°. The diffraction patterns were compared with those reported in the Inorganic Crystal Structure Database (ICSD).

Transmission electron microscopy (TEM) images of samples were observed using a JEOL 1400 electron microscope operating at 80–120 kV, and the size of the particles was estimated using ImageJ software. The high-resolution transmission electron microscopy (HR-TEM), high-angle annular dark-field imaging (HAADF), and energy dispersive X-ray spectroscopy (EDS) images were acquired using an FEI (S)TEM Titan G2 600–300 Hitachi HT7700 microscope at an accelerating voltage of 80 kV to analyze the morphology and element distribution in the NPs.

DLS was performed for the synthesized core@shell NPs in hexane and surface-modified NPs dispersed in water with a Malvern Zetasizer Nano ZS instrument. IR spectra were recorded using an FT-IR spectrophotometer, JASCO 4200, in transmission mode.

The spectroscopic characterization of the NPs in the form of powders and ligand-free NPs dispersed in water or whole human blood (1 mg·mL^–1^) was done at room temperature using a fiber-coupled CNI 2 W continuous wave diode laser with 808-, 975-, 1208-, and 1532 nm wavelengths equipped with a PIXIS:256E Digital CCD Camera and SP-2156 Imaging Spectrograph (Princeton Instruments). A 10A-PPS power meter (Ophir Photonics) determined the beam size and laser powers.

The excitation spectra were measured by using an Opolette 355LD UVDM tunable pulsed laser with a repetition rate of 20 Hz (OPOTEK Inc.) and a QuantaMaster 40 spectrophotometer (Photon Technology International) equipped with a R928 photomultiplier (Hamamatsu).

Temperature-dependent emissions of the powdered samples were measured by exciting samples with a 1532 nm 50 mW diode laser placed in a tubular electric furnace (Gero RES-E 230/3), where the temperature of the sample was controlled *via* a type K thermocouple in contact with it. The emissions from the oven were focused on the entrance slit of a spectrograph (Andor SR-303i-A) equipped with a cooled CCD camera (Andor Newton). The measurements of temperature-dependent emission of the UCNPs in the blood were taken in the temperature-controlled holder for cuvettes with a PIXIS:256E Digital CCD Camera and SP-2156 Imaging Spectrograph used for emission collection. All spectra were corrected for the equipment’s spectral response. A 10A-PPS power meter (Ophir Photonics) determined the beam size and laser powers.

## 3 Results and Discussion

### 3.1 Structure and Morphology of the Synthesized NPs

**Figure 1:**
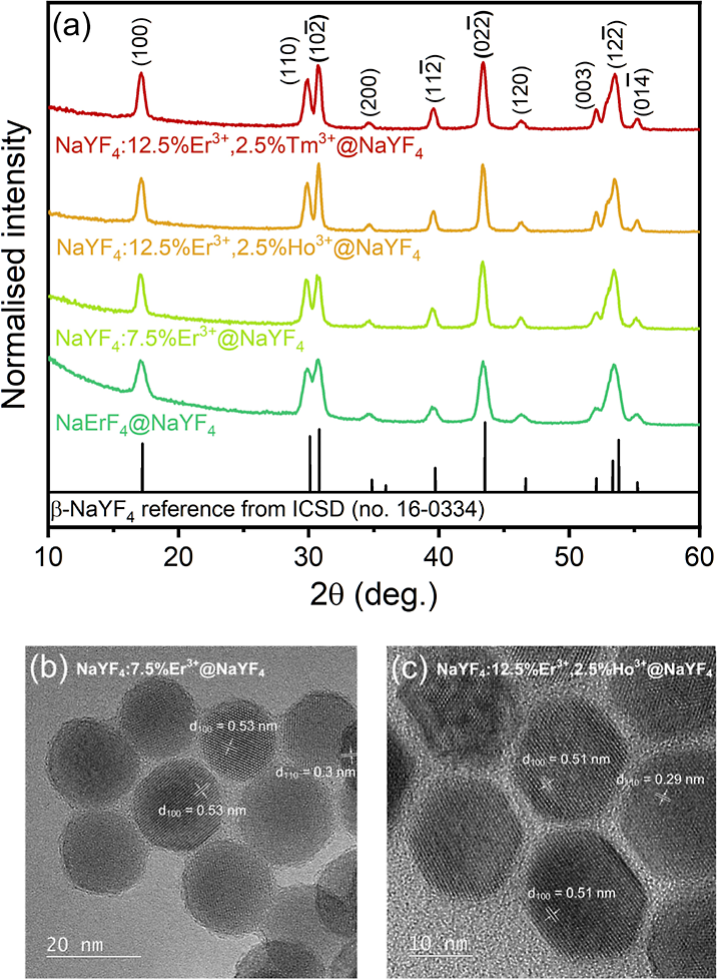
(a) XRD patterns of the synthesized core@shell NPs compared with reference from ICSD database no. 16-0334. HR-TEM images of (b) NaYF_4_:7.5% Er^3+^@NaYF_4_ and (c) NaYF_4_:12.5% Er^3+^, 2.5% Ho^3+^@NaYF_4_ NPs.

The synthesized NPs were thoroughly analyzed to confirm their desired compositions and morphology. The structure and phase purity of the NPs were determined by XRD analysis (Figure 1a). The products exhibited a high crystallinity and ordered hexagonal phase consistent with the reference pattern of β-NaYF_4_ from the ICSD database (no. 16-0334). The shape and peak separation correlate with the size of NPs; *i.e.*, the broader the XRD peaks, the smaller the NPs. This observation is consistent with the TEM analysis presented in Figures 1b,c and S1.

The TEM images of NPs were collected to determine their size distribution and morphology (Figures 1b,c and S1). The synthesis of NaErF_4_@NaYF_4_ and NaYF_4_:7.5% Er^3+^@NaYF_4_ allowed us to obtain the spherical particles of 12.7 ± 1.8 and 17.7 ± 1.8 nm, respectively. In contrast, the NaYF_4_:12.5% Er^3+^, 2.5% Ho^3+^@NaYF_4_ and NaYF_4_: 12.5% Er^3+^, 2.5% Tm^3+^@NaYF_4_ NPs are in the form of small oval-shaped NPs with slightly larger average width: 20.2 ± 1.7 and 22.8 ± 2.2 nm, respectively. The synthesized crystals were of good quality, as evidenced by their narrow size distribution and HR-TEM images in Figures 1 and S1. The differences between sizes result from different compositions of the NPs. The obtained results indicate that doping with Ho^3+^ and Tm^3+^ affected the size and shape of prepared NPs despite the chemical similarity between the used RE^3+^ ions. HR-TEM images also revealed clear lattice fringes with spacings of 0.53 and 0.51 nm corresponding to the (100) planes and 0.3 and 0.29 nm corresponding to the (110) planes of hexagonal NaYF_4_.

Two of the synthesized types of NPs, *i.e.*, NaYF_4_:7.5% Er^3+^@NaYF_4_ and NaYF_4_:12.5% Er^3+^, 2.5% Ho^3+^@NaYF_4_, were subjected to EDS elemental mapping (Figure S2). The analysis indicated a homogeneous distribution of Y^3+^ ions in the NPs, while Er^3+^ concentrated in the centers of NPs. These results prove the formation of the core@shell structures.

**Figure 2:**
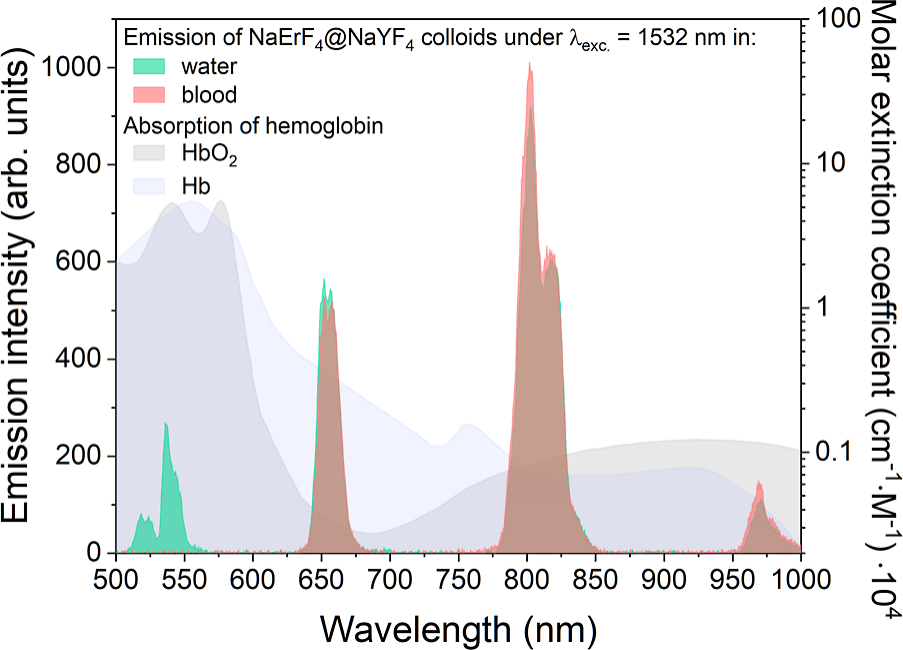
UC luminescence of NaErF_4_@NaYF_4_ NPs measured under 1532 nm laser excitation (with a power density of 5 W·cm^–2^) in both water and full human blood colloids, each with NP concentration of 1 mg·mL^–1^ and correlated with hemoglobin absorption.

The surface modification of NaErF_4_@NaYF_4_ and NaYF_4_:7.5% Er^3+^@NaYF_4_ NPs was confirmed by a zeta potential measurement. Removing OA from the NPs’ surface resulted in positively charged (40.2 ± 7.9 mV) and hydrophilic NPs. The FT-IR spectra measurements of OA-capped and ligand-free NaErF_4_@NaYF_4_ were also registered (Figure S3), confirming a significant reduction of bands related to organic groups stretching vibrations because of dissociation of OA from the NPs’ surface. We have selected NaErF_4_@NaYF_4_ and NaYF_4_:7.5% Er^3+^@NaYF_4_ NPs to study them in blood as they presented the highest luminescence intensity band at 984 nm, which we expected to be also intense in the blood. The NaErF_4_@NaYF_4_ NPs were used only for studies presented in Figure 2, while the emission of NaYF_4_:7.5% Er^3+^@NaYF_4_ NPs was measured at different temperatures as presented in Figure 6.

### 3.2 Spectroscopic Properties of NaErF_4_@NaYF_4_ NPs in Whole Blood

The luminescent properties of the synthesized NPs depend strongly on their composition and temperature. However, before discussing the received spectra as a function of temperature, it is important to understand why luminescent thermometers based on LIR of nonthermally coupled levels may be useful, especially for applications in a biological medium, such as whole human blood or, in general, in studies *in vivo*. In the most studied systems in which Er^3+^ ions are applied as luminescent centers, the emission from thermally coupled levels of Er^3+^ ions, *i.e.*, the ratio between ^2^H_11/2_ → ^4^I_15/2_ and ^4^S_3/2_ → ^4^I_15/2_ at 520 and 545 nm is used for optical thermometry purposes.^51−53^ However, as indicated in Figure 2, their usefulness in systems involving blood, specifically hemoglobin, is limited as only radiation with a wavelength above 600 nm can be detected.^54^

Figure 2 compares the UC spectra of NaErF_4_@NaYF_4_ NPs registered in water and whole human blood under excitation with a 1532 nm NIR laser. Because of the absorption of hemoglobin, the green bands at around 520 and 545 nm are quenched, and only bands related to ^4^F_9/2_ → ^4^I_15/2_, ^4^I_9/2_ → ^4^I_15/2_, and ^4^I_11/2_ → ^4^I_15/2_ at 661, 813, and 984 nm can be observed. Therefore, the luminescent bands that can be used for LIR determination as a function of temperature are only those between 600 and 1000 nm. This range is slightly broader than would be inferred from the literature’s first BW range.^26^ In this context, it should also be emphasized that using the typical 980 nm excitation wavelength for UC further restricts this range. Therefore, we decided to use the excitation wavelength from the third BW range, *i.e.*, 1532 nm, for the studies provided in this article. This wavelength allows for better penetration of human blood, as we have previously demonstrated,^54^ but also enables the detection of the Er^3+^ luminescent band at 984 nm. This feature provides additional opportunities for determining the LIRs that offer the best temperature sensitivity.

### 3.3 Processes behind the Upconversion of NPs Sensitized by Er^3+^ Ions

**Figure 3:**
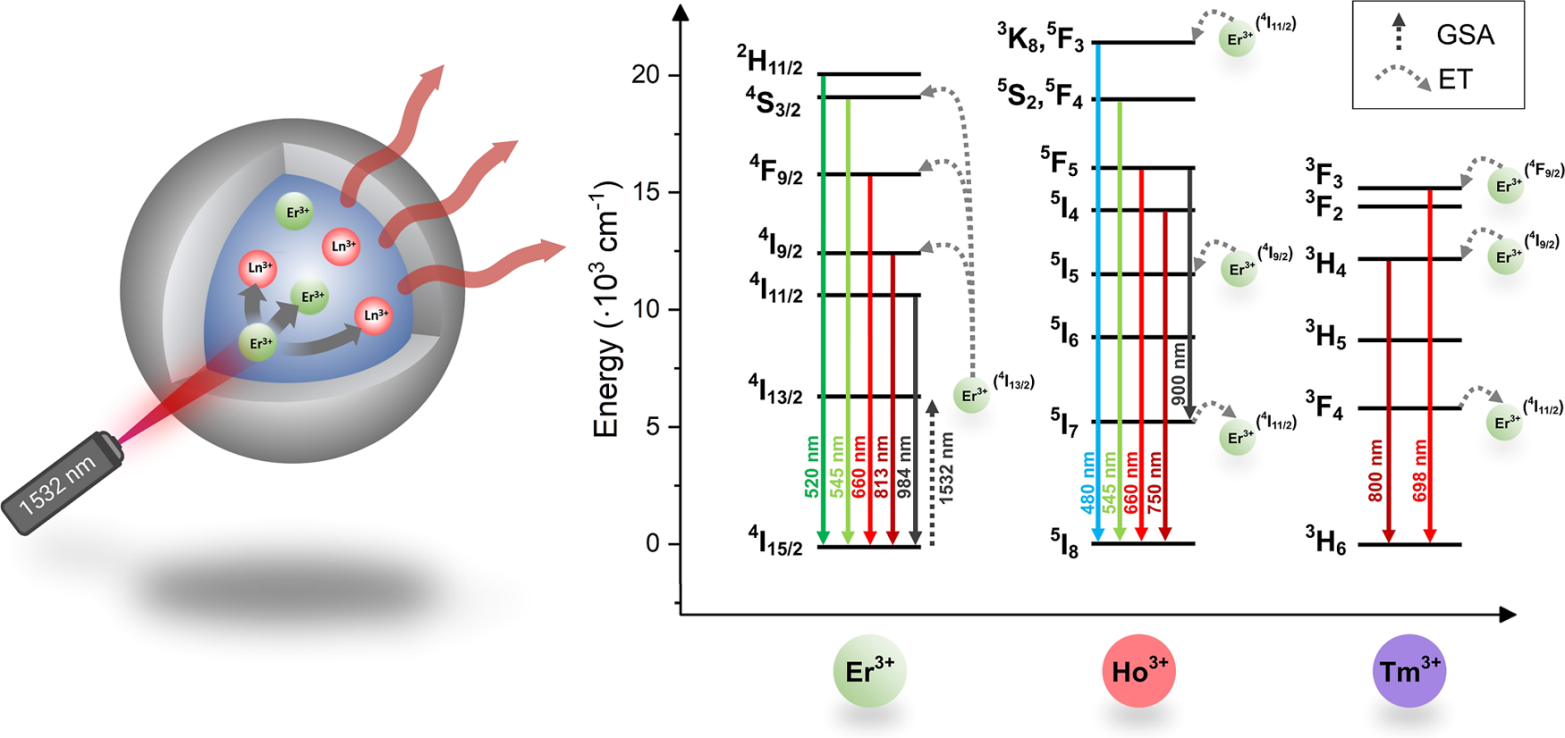
Simplified mechanism of photon UC in NPs under 1532 nm laser radiation doped with Er^3+^ ions or double-doped Er^3+^/Ho^3+^ and Er^3+^/Tm^3+^. The Er^3+^ ions undergo GSA under 1532 nm radiation, and most of them are in the ^4^I_13/2_ excited state and serve as energy donors to other Er^3+^ ions. Ho^3+^ and Tm^3+^ ions can be excited *via* ET from the Er^3+^ ions. From the many possible pathways of ET, in the image, two Er^3+^ → Ho^3+^ ET are proposed: (I) Er^3+^(^4^I_9/2_) + Ho^3+^(^5^I_8_) → Er^3+^(^4^I_15/2_) + Ho^3+^ (^5^I_5_) and (II) Er^3+^(^4^I_9/2_) + Ho^3+^(^5^I_6_) → Er^3+^(^4^I_15/2_) + Ho^3+^(^3^K_8_,^5^F_3_).^61^ In the case of Er^3+^ → Tm^3+^ ET, we propose two ways of ET: (I) Er^3+^(^4^I_9/2_) + Tm^3+^(^3^H_6_) → Er^3+^(^4^I_15/2_) + Tm^3+^(^3^H_4_) and (II) Er^3+^(^4^F_9/2_) + Tm^3+^(^3^H_6_) → Er^3+^(^4^I_15/2_) + Tm^3+^(^3^F_3_). In the double-doped system, the Ho^3+^ at ^5^I_7_ and Tm^3+^ at ^3^F_4_ transfer energy to Er^3+^ at their ^4^I_11/2_ excited state, enhancing the red emission of Er^3+^ ions.^5,62^

Er^3+^-doped NPs can be excited by 808, 975, and 1532 nm laser radiation *via* the sequence of GSA and ESA processes followed by cross-relaxation (CR) between Er^3+^ ions (see Figure S4 for the excitation spectra of NaErF_4_@NaYF_4_ NPs).^33,39^ Here, we discuss the possibility of UC under 1532 nm excitation based on the sensitizing properties of Er^3+^ ions and the ET between them and other dopants, *i.e.*, Ho^3+^ and Tm^3+^ ions. Co-doping NPs with other Ln^3+^ ions allows obtaining additional emission bands in the first BW. The simplified mechanism of the processes occurring in the studied NPs is presented in Figure 3. At this point, we are not discussing the exact mechanisms occurring in the examined samples. Instead, our focus is solely on the effects related to the use of the obtained NPs as temperature sensors. More information about the processes taking place in the studied systems can be found in published research, *e.g.*, about Er^3+^-sensitized UC,^33,43,55−58^ Er^3+^/Ho^3+^ system,^40,57,59^ and Er^3+^/Tm^3+^ interactions.^33,55,59,60^

Er^3+^ ions in NaErF_4_@NaYF_4_ and NaYF_4_:7.5% Er^3+^@NaYF_4_ NPs undergo excitation *via* GSA, *i.e.*, ^4^I_15/2_ → ^4^I_13/2_ electronic transition when irradiated by a 1532 nm laser (see Figure 3). The excited Er^3+^ ions can transfer the absorbed energy to other Er^3+^ ions in their ^4^I_13/2_ excited state (*via* ET upconversion, ETU). Also, other ETs are possible, as depicted in Figure 3, finally yielding Er^3+^ ions in their ^4^S_3/2_ excited state and, due to the thermalization process, to the ^2^H_11/2_ excited state. Typically, the high concentration of Er^3+^ in NPs yields more intense emission as these ions undergo a self-sensitization process.^33^ Also, shifting the emission color from green to red due to CR processes usually occurs together with changes in the emission intensity.^33,39^ The excited Er^3+^ ions emit the absorbed energy by typical radiative transitions, *i.e.*, ^2^H_11/2_ → ^4^I_15/2_ (520 nm), ^4^S_3/2_ → ^4^I_15/2_ (545 nm), ^4^F_9/2_ → ^4^I_15/2_ (660 nm), ^4^I_9/2_ → ^4^I_15/2_ (813 nm), and ^4^I_11/2_ → ^4^I_15/2_ (984 nm).

The proposed UC mechanism is confirmed by the measured dependencies of emission intensity on laser power density (see Figure S5 and eq S1). The emission of Er^3+^ ions from the ^4^S_3/2_ excited state is three photonic as the integral luminescence intensity dependence on power density in the diagram with double logarithmic scale has a slope slightly exceeding two for NaErF_4_@NaYF_4_ and NaYF_4_:7.5% Er^3+^@NaYF_4_ NPs, respectively. The lower than theoretical number of photons required to observe UC indicates that other processes occur in the studied NPs, such as CR, and ET to other ions. The emissions from ^4^I_9/2_ and ^4^I_11/2_ of Er^3+^ ions related to the bands at 813 and 984 nm are two photons, as confirmed by the data presented in Figure S5, which is also consistent with the proposed model.

**Figure 4:**
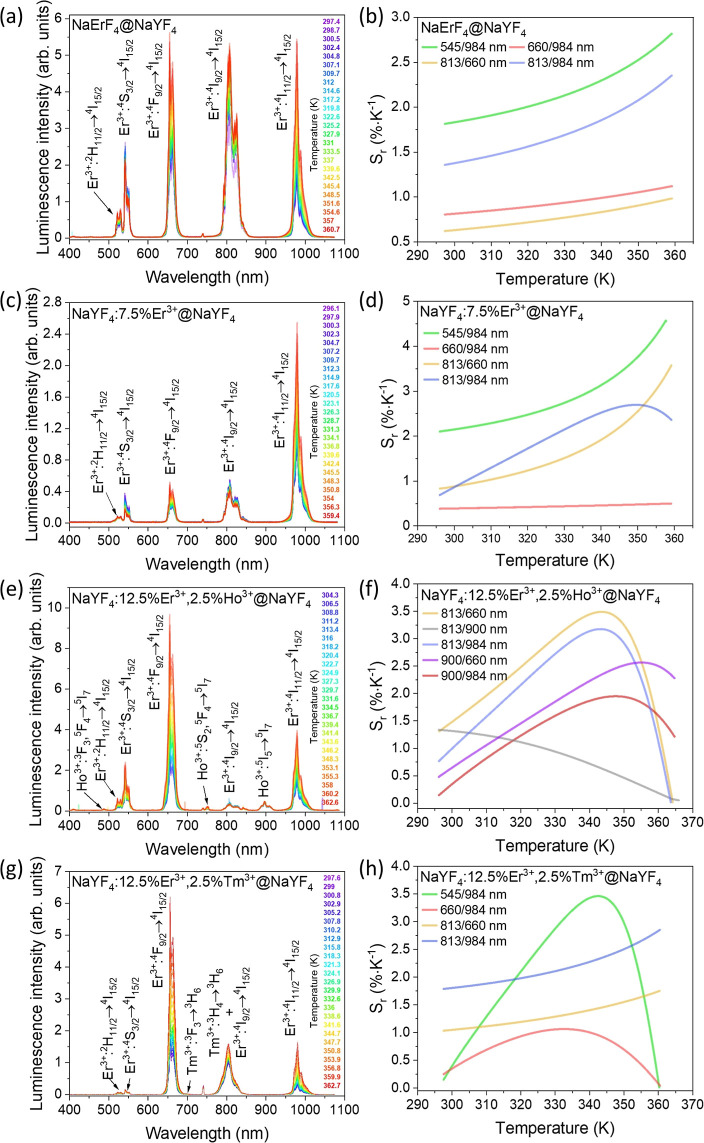
(a,c,e,g) Temperature-dependent emission spectra of the synthesized NPs measured under laser 1532 nm excitation and the power density of 25 W·cm^–2^ with electron transitions of dopant ions assigned to respective bands. (b,d,f,h) Relative temperature sensitivities determined from the collected emission spectra and the LIRs between the observed emission bands (see Figure S6 for the LIRs).

The Ho^3+^ ions in the NaYF_4_:12.5% Er^3+^, 2.5% Ho^3+^@NaYF_4_ NPs are excited by ET from Er^3+^, as demonstrated in Figure 3. From the many possible pathways of ET, here we postulate that Ho^3+^ ions are populated to their ^5^I_5_ excited state *via* ET from Er^3+^ ions in their ^4^I_9/2_ excited state as the energy between these two levels is similar. Next, Ho^3+^ ions can undergo another ET from Er^3+^ ions in their ^4^I_9/2_ state to reach ^5^S_2_, ^5^F_4_, or ^3^K_8_,^5^F_3_ levels, allowing for emissions at 480, 545, and 900 nm, respectively (after relaxation). The emission of Er^3+^ ions at 813 nm, related to the ^4^I_9/2_ → ^4^I_15/2_ transition, is lowered in the presence of Ho^3+^ ions, confirming our assumption. However, the results indicate that two photons are necessary to populate the ^5^F_5_ excited state of Ho^3+^ ions, which is much lower than expected. Considering that the emission of Ho^3+^ at 900 nm visible in Figure 4e is relatively low in comparison to the emission of Er^3+^ ions, the explanation for the lowered number of photons than assumed is that the ET and radiative depopulation processes compete with CRs and other nonradiative routes of Ho^3+^ excited-state depopulation. The increased emission at 660 nm of Er^3+^ ions in the presence of Ho^3+^ ions confirms ET from Ho^3+^ in their ^5^I_7_ excited state to Er^3+^ ions in their ^4^I_11/2_ excited state.^62^ The mechanism of Er^3+^ to Ho^3+^ ET was the subject of several studies and can vary depending on the composition and type of material.^61,63,64^

The NaYF_4_:12.5% Er^3+^, 2.5% Tm^3+^@NaYF_4_ NPs present an intense red UC connected with the ^4^F_9/2_ → ^4^I_15/2_ transition of Er^3+^ ions (see Figure 4g). Additionally, the emission band at 800 nm is much wider than that in Er^3+^-only doped NPs, which indicates the emission of Tm^3+^ related to the ^4^H_4_ → ^3^H_6_ transition. In Figure 4g, a small emission band at around 698 nm is also visible, related to the ^3^F_3_ → ^3^H_6_ transition of the Tm^3+^ ions. Those emission properties of Er^3+^ and Tm^3+^-doped NPs are a result of ET from Er^3+^ to Tm^3+^ ions *via* Er^3+^(^4^I_9/2_) + Tm^3+^(^3^H_6_) → Er^3+^(^4^I_15/2_) + Tm^3+^(^3^H_4_) and Er^3+^(^4^F_9/2_) + Tm^3+^(^3^H_6_) → Er^3+^(^4^I_15/2_) + Tm^3+^(^3^F_3_) routes.^33,65^ Additionally, the presence of Tm^3+^ ions has a great influence on the population of the ^4^F_9/2_ excited state of Er^3+^ ions as they transfer back part of the energy by the Tm^3+^(^3^F_4_) + Er^3+^(^4^I_11/2_) → Tm^3+^(^3^H_4_) + Er^3+^(^4^F_9/2_) process, thus shifting the green emission of Er^3+^ ions to red.^5,33^ This is in line with the determined number of photons responsible for the UC of Er^3+^ ions at 660 nm, which is higher than the number of other NPs studied. The remaining emission bands are mainly a result of Er^3+^ electronic transitions; therefore, the number of photons determined for each emission band is similar to that of all of the studied NPs.

The UC of the NPs presented here under excitation by a 1532 nm laser is intense, and the best-emitting sample shows an emission intensity similar to that of NaYF_4_:18% Yb^3+^, 2% Er^3+^@NaYF_4_ NPs, which are known as compounds with one of the best UC efficiencies. Figure S10 presents a comparison of the emission from NPs excited by a 1532 nm laser with the emission from NaYF_4_:18% Yb^3+^, 2% Er^3+^@NaYF_4_, which has a known UCQY under excitation by a 975 nm laser with the same power density as at 1532 nm, equal to 0.67 ± 0.11%.^49^ The comparison indicates that the NaErF_4_@NaYF_4_ NPs, which are the best emitters among the four whose properties we present, have a similar emission intensity to NaYF_4_:18% Yb^3+^, 2% Er^3+^@NaYF_4_, and therefore, the estimated UCQY is also similar. Table S2 presents the estimated UCQY values obtained based on the reference sample with a known quantum yield.

### 3.4 Temperature-Dependent Upconversion of NPs

The luminescence of studied NPs under 1532 nm excitation was sensitive to the temperature and highly dependent on the composition. Figure 4a,c,e,g demonstrates the collected emission spectra, and Figure S6a,c,e,g presents the integrated emission intensity of the determined emission bands. In most cases, the intensity of emission peaks increased with the growing temperature of measurement, with some exceptions, *i.e.*, the 545 nm emission band of Er^3+^ ions in NaErF_4_@NaYF_4_ and NaYF_4_:7.5% Er^3+^@NaYF_4_ as well as emission of these ions at 813 nm in the case of NaYF_4_:7.5% Er^3+^@NaYF_4_ and NaYF_4_:12.5% Er^3+^, 2.5% Ho^3+^@NaYF_4_ NPs. The decrease in the former results from the thermalization of the ^2^H_11/2_ excited state at a higher temperature.^66^ The UC intensity of Er^3+^ ions and the ratio between emission bands highly depended on their concentration. Higher concentrations of Er^3+^ ions favor better absorption of the excitation radiation and introduce a self-sensitization effect.^33,55,67^

Introducing Ho^3+^ or Tm^3+^ ions into NPs’ structure changed the emission properties of Er^3+^ ions, mainly enhanced red emission at 660 nm, which is an advantage considering signal collection in biological medium.^68^ The overall emission intensity of NaYF_4_:12.5% Er^3+^, 2.5% Ho^3+^@NaYF_4_ and NaYF_4_:12.5% Er^3+^, 2.5% Tm^3+^@NaYF_4_ NPs was similar to the emission of NPs doped with only Er^3+^ ions, which indicates that any potential quenching resulting from Er^3+^-Ho^3+^ and Er^3+^-Tm^3+^ interactions is relatively minimal. The presence of Ho^3+^ ions in the NPs allowed for the observation of two additional emission bands at 750 and 900 nm, from which the second one, related to the ^5^I_5_ → ^5^I_7_ transition, only slightly changes with temperature, which is promising for temperature sensing *via* the LIR technique.

Tm^3+^ ions in the NaYF_4_:12.5% Er^3+^, 2.5% Tm^3+^@NaYF_4_ NPs modified the energy processes occurring in these NPs under 1532 nm excitation, which resulted in quenching the green emission of Er^3+^ ions and an increase of red emission as mentioned above. Additionally, the presence of Tm^3+^ ions affected the shape and intensity of the emission band at 813 nm, which resulted from the typical emission of these ions related to the ^3^H_4_ → ^3^H_6_ electronic transition.^69^ The enhancement of the NIR band at 813 nm was one of the present research’s goals as it falls within the first BW.

To calculate the LIRs and *S*_r_ values, we used the approach described in the Supporting Information section (eqs S2–S5). The determined LIRs are presented in Figure S6g,d,f,h. From all the calculated maximum *S*_r_ values, the highest were determined for 545/984 and 813/984 nm LIRs in the case of NaErF_4_@NaYF_4_ and NaYF_4_:12.5% Er^3+^, 2.5% Tm^3+^@NaYF_4_ NPs; 545/984 and 813/660 nm LIRs of NaYF_4_:7.5% Er^3+^@NaYF_4_ NPs; and 813/660 and 813/984 nm LIRs in the case of NaYF_4_:12.5% Er^3+^, 2.5% Ho^3+^@NaYF_4_ NPs. Table S1 in the Supporting Information collects all of the maximum *S*_r_ values. The highest *S*_r_ value equal to 4.55%·K^–1^ at 359 K was determined for the 545/984 nm LIR of NaYF_4_:7.5% Er^3+^@NaYF_4_ NPs. The best performance as a nanothermometer in the first BW can be expected from the NaYF_4_:12.5% Er^3+^, 2.5% Ho^3+^@NaYF_4_ NPs, which 813/660 nm LIRs allowed to calculate maximum *S*_r_ equal to 3.48%·K^–1^ at 343 K and 1.93%·K^–1^ at 310 K (temperature of human body). The determined *S*_r_ value is among the highest reported for UC-based nanothermometers used in biological systems.^30^

Figure S8 shows the temperature uncertainties determined for the studied NPs, based on 100 measurements at room temperature. The NaYF_4_:7.5% Er^3+^@NaYF_4_ and NaYF_4_:12.5% Er^3+^, 2.5% Ho^3+^@NaYF_4_ NPs presented the lowest values for 813/984 LIR, *i.e.*, 0.05 and 0.06 K, respectively. Meanwhile, Figure S9 presents the changes in LIRs between the 813 and 660 nm bands from 297 to 353 K as a function of cycle number. The measurements show that the changes in LIRs between these two temperatures are repeatable, and the obtained values are similar to each other in each of the 10 heating–cooling cycles of the NPs.

Besides the LIRs typical for the BWs range, we also investigated the thermometric properties of the studied NPs, focusing on their characteristic green emission and thermally coupled bands at 520 and 545 nm, obtained under 1532 nm laser irradiation (see Figure S7). Interestingly, one of the samples, specifically the NaYF_4_:7.5% Er^3+^@NaYF_4_ sample, demonstrated a relative sensitivity of 2.19%·K^–1^ at 298 K, which is higher than the typical results published.^53,70^

**Figure 5:**
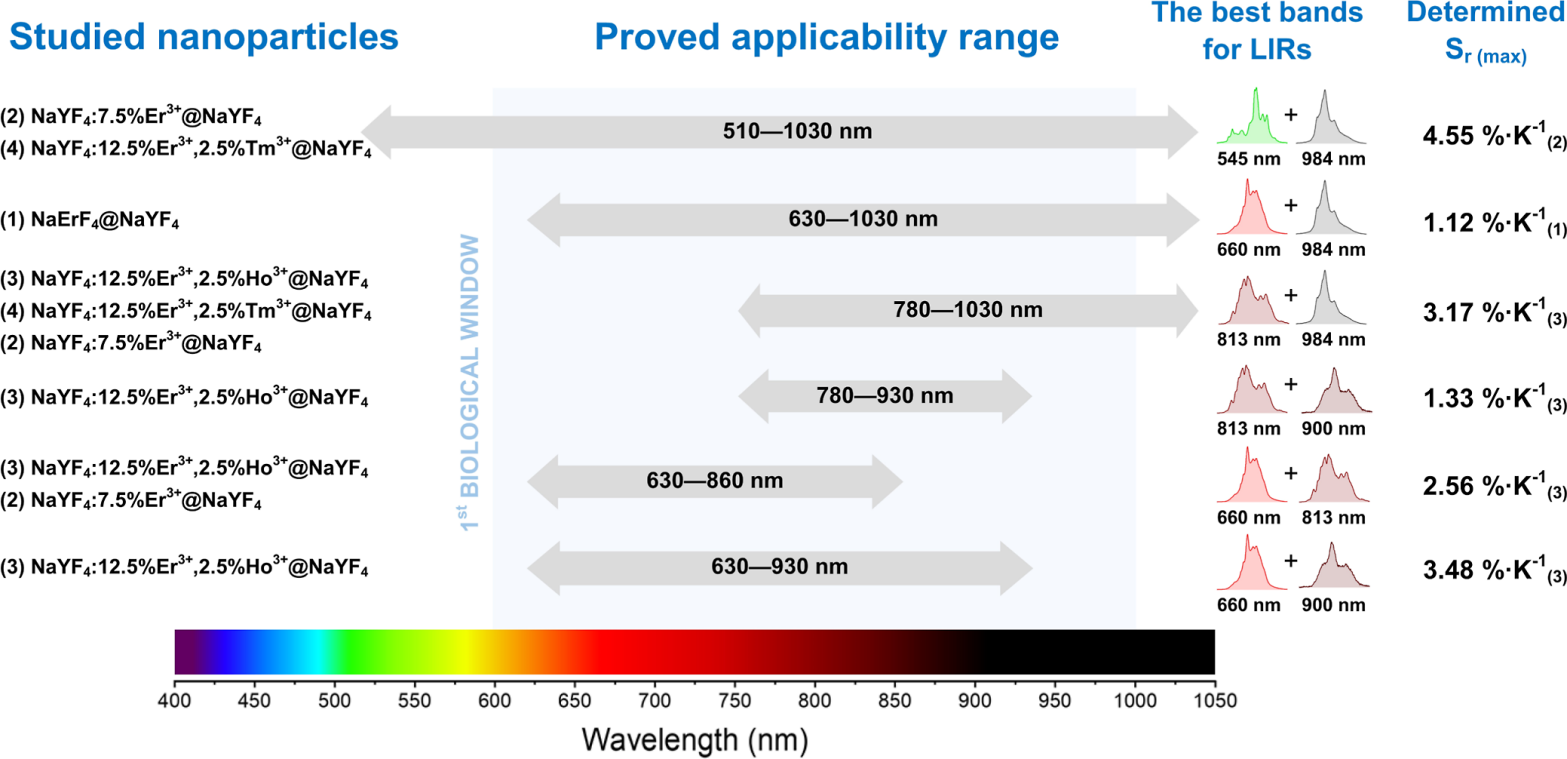
Overview facilitating the synthesis of experimental findings. The left panel presents the NPs employed in the study (1–4); the central segment illustrates the spectral range wherein the luminescence of NPs may be used for temperature detection, juxtaposed with the spectral range of the 1st BW (depicted by the gray background). The right part demonstrates the emission bands viable for temperature monitoring within the specified spectral range, accompanied by the maximum relative sensitivities achieved for a given NP (in the subscript). The remaining maximum *S*_r_ values, as well as those determined for human body temperature, are presented in Table S1, along with the absolute sensitivities, *S*_a_.

**Figure 6:**
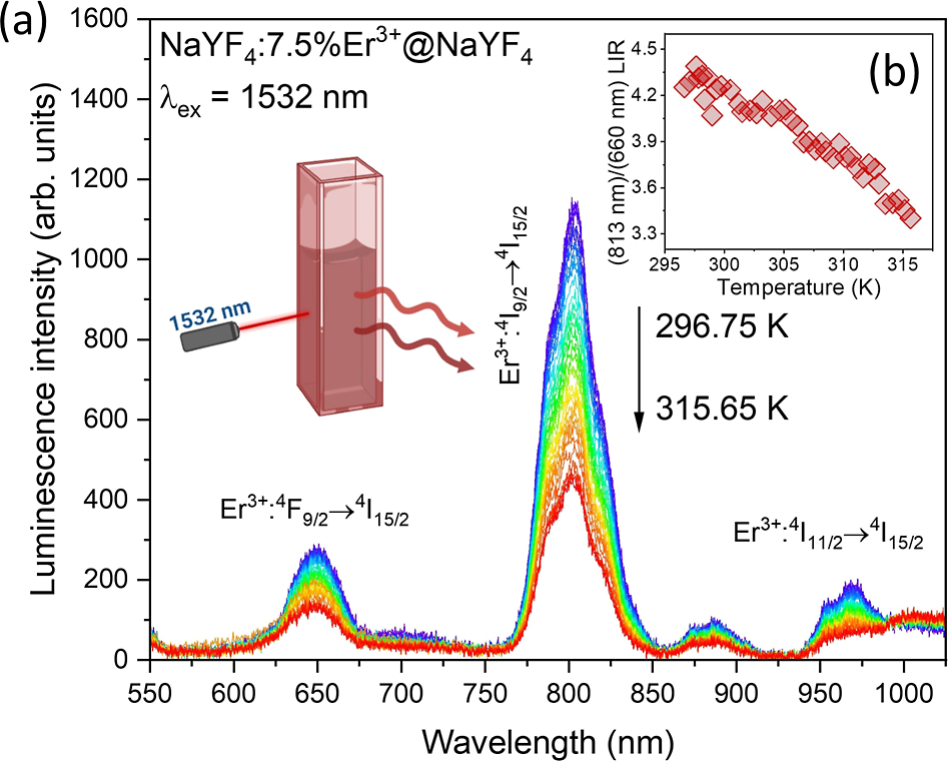
(a) Temperature-dependent UC emission of NaYF_4_:7.5% Er^3+^@NaYF_4_ NPs measured under 1532 nm laser excitation (with a power density of 5 W·cm^–2^) in whole human blood and NPs concentration of 1 mg·mL^–1^. (b) LIR between the 813 and 660 nm emission bands calculated from the collected emission spectra.

To better illustrate the achievements/discoveries obtained as a result of the studies, we have compiled the obtained data in the form of a scheme in Figure 5. Thanks to such a presentation of the results, it is possible to select appropriate NPs depending on the spectral range in which the temperature is to be investigated. For example, to determine the temperature in an environment containing whole blood, we can use the 630–860 nm range, in which blood exhibits relatively good transparency (see Figure 2) and emission bands at 660 and 813 nm. For such studies, NPs nos. (2) and (3) (Figure 5) exhibit the best characteristics, namely, NaYF_4_:7.5% Er^3+^@NaYF_4_ and NaYF_4_:12.5% Er^3+^, 2.5% Ho^3+^@NaYF_4_, with the latter showing the best sensitivity in the given range. In our experiments with whole human blood, we used NPs no. (2) to test whether LIR measurement is possible, which ended successfully (see Figure 6).

### 3.5 Temperature Measurement in Whole Human Blood

Whole human blood is an exceptionally challenging environment for studying the luminescence of the NPs contained within it. Due to absorption by hemoglobin, the luminescent properties of NPs must be appropriately selected, *i.e.*, their luminescence must be intense, and emission bands must fall within the first BW range. Additionally, NPs must be susceptible to being transferred into water and form stable colloids. It is also important to note that blood is sensitive to temperature changes and quickly coagulates without a stabilizer such as EDTA. In our experiment, we used freshly drawn blood from a donor, and as test NPs, we employed NaYF_4_:7.5% Er^3+^@NaYF_4_. During the measurement, the blood was continuously mixed, and the power density of the excitation laser (5 W·cm^–2^) was experimentally determined beforehand as the highest without causing changes in the studied system.

As seen in Figure 6, emission under the influence of a 1532 nm laser could be registered. In one of our previous works, we proved that this wavelength is the best for spectroscopic studies of NPs in blood among four other wavelengths tested, *i.e.*, 808, 975, 1208, and 1532 nm.^54^ The emission shown in Figure 6 differs from that recorded for NPs in powder form, as visible in Figure 4c. This is due to the modification of NPs, *i.e.*, the removal of OA molecules from their surface and dispersion in blood. However, this does not change the fact that the ratio of the 813 to 660 nm bands depends on the system’s temperature and can be used for temperature registration. The revealed properties require prior calibration of such a system before it can be used as a reliable nanothermometer.

## 4 Summary and Conclusions

Upconverting NPs doped with Ln^3+^ ions are extensively investigated because of their unique luminescent properties, making them valuable for various applications, including sensors for temperature, as studied in this article. One of the most important features of upconverting NPs is their ability to emit and be excited within BWs, where biological materials are more transparent to radiation. NPs doped with Er^3+^ ions studied here can undergo UC under NIR laser excitation at 1532 nm and emit within the 520–1000 nm range.

We have synthesized core@shell NPs, specifically NaErF_4_@NaYF_4_, NaYF_4_:7.5% Er^3+^@NaYF_4_, NaYF_4_:12.5% Er^3+^, 2.5% Ho^3+^@NaYF_4_, and NaYF_4_:12.5% Er^3+^, 2.5% Tm^3+^@NaYF_4_ to study their luminescent properties. These NPs were prepared using reactions in high boiling solvents (OA and octadecene) and had the desired structure, high crystallinity, and narrow size distribution. Elemental mapping showed a homogeneous Y^3+^ and Er^3+^ ion distribution, verifying the core@shell structure.

The luminescence behavior of the NPs was studied under 1532 nm excitation. Temperature-dependent emission properties were analyzed within the 295–360 K range, showing that the emission intensity and band ratios varied with the temperature, indicating their potential for temperature sensing.

The present study explored the UC mechanisms involving Er^3+^ ions and their ET to Tm^3+^ or Ho^3+^ codopants. These processes lead to various emission bands, including green, red, and NIR luminescence, depending on the NPs’ composition. Codoping with Ho^3+^ and Tm^3+^ introduced additional emission bands within the first BW, *i.e.*, at 900 nm (Ho^3+^) and around 800 nm (Tm^3+^). The maximum relative temperature sensitivity of the NPs ranged from 4.55%·K^–1^ to 1.12%·K^–1^, with temperature resolution between 0.05 and 2.53 K at room temperature. The composition of the NPs and the specific luminescent bands used for the luminescence ratio calculations influenced these properties. NaYF_4_:7.5% Er^3+^@NaYF_4_ NPs presented the best relative sensitivity among all the studied samples, *i.e*., 4.55%·K^–1^ for 545/984 nm LIR. However, NaYF_4_:12.5% Er^3+^, 2.5% Ho^3+^@NaYF_4_ NPs presented the best properties for temperature sensing in biorelated applications because of high maximum relative sensitivities within red and NIR range, *i.e.*, 3.48%·K^–1^ for 813/660 nm LIR, 3.17%·K^–1^ for 813/984 nm LIR, 2.56%·K^–1^ for 900/660 nm LIR, and 1.94%·K^–1^ for 900/984 nm LIR.

Our study also demonstrated the potential of NaYF_4_:7.5% Er^3+^@NaYF_4_ NPs for temperature sensing in biological environments. Under 1532 nm excitation, the NPs’ luminescence was measured in whole human blood, showing that the ratio of Er^3+^ ion emission bands changes with temperature. It is a promising result, allowing the development of Er^3+^-sensitized nanothermometers in the future.

The results indicate that Er^3+^-sensitized NPs can be ultrasensitive and adjustable thermometers within BWs. These NPs have promising applications in nanomedicine for noncontact temperature sensing.

## Supporting Information Available

TEM images and size distributions of all obtained NPs; HAADF images and EDS maps of distribution measured for NaYF_4_:7.5% Er^3+^@NaYF_4_ and NaYF_4_:12.5% Er^3+^, 2.5% Ho^3+^@NaYF_4_ NPs; FT-IR spectra of OA-capped and ligand-free NaErF_4_@NaYF_4_ NPs; excitation spectra of NaErF_4_@NaYF_4_ NPs; the methodology used for calculation of the number of photons responsible for the observed upconversion; dependencies of luminescence intensity on 1532 nm laser power density measured for all of the obtained NPs; methodology used for the calculation of temperature sensing parameters; dependences of integrated emission bands intensity on temperature; LIRs calculated based on the collected emission spectra as a function of temperature; calculated absolute and relative temperature sensitivities of NPs; temperature uncertainties calculated based on 100 measurements of emission spectra, the changes in LIRs between the 813 and 660 nm bands from 297 to 353 K as a function of cycle number; and comparison of the luminescence of the obtained NPs to the emission of NaYF_4_:18% Yb^3+^, 2% Er^3+^@NaYF_4_ NPs with known UCQY (PDF)
