# Supplementary material for: Ultrasensitive and Adjustable Nanothermometers Based on Er3+-Sensitized Core@Shell Nanoparticles for Use in the First Biological Window
Source: ACS Appl Mater Interfaces. 2024 Oct 4;16(41):55925–35. doi: 10.1021/acsami.4c10176 (PMC11492177; doi:10.1021/acsami.4c10176)
Supplement: Supplementary file 1 — am4c10176_si_001.pdf [file am4c10176_si_001.pdf]

Supporting Information

Ultra-sensitive and adjustable nanothermometers based on Er^{3+}-sensitized core@shell nanoparticles for use in the first biological window

Tomasz Grzyb[1*], Sylwia Ryszczyńska[2], Natalia Jurga[1], Dominika Przybylska[1], Inocencio R. Martín[3]

[1]Department of Rare Earths, Faculty of Chemistry, Adam Mickiewicz University in Poznań, Uniwersytetu Poznańskiego 8, 61-614 Poznań, Poland

[2]Department of Chemistry, Faculty of Forestry and Wood Technology, Poznan University of Life Sciences, Wojska Polskiego 75, 60-625 Poznań, Poland

[3]Departamento de Fisica, Instituto de Materiales y Nanotecnología (IMN), Universidad de La Laguna, 38200 San Cristóbal de La Laguna, Santa Cruz de Tenerife, Spain

*Correspondence: tgrzyb@amu.edu.pl

1. Structure and morphology

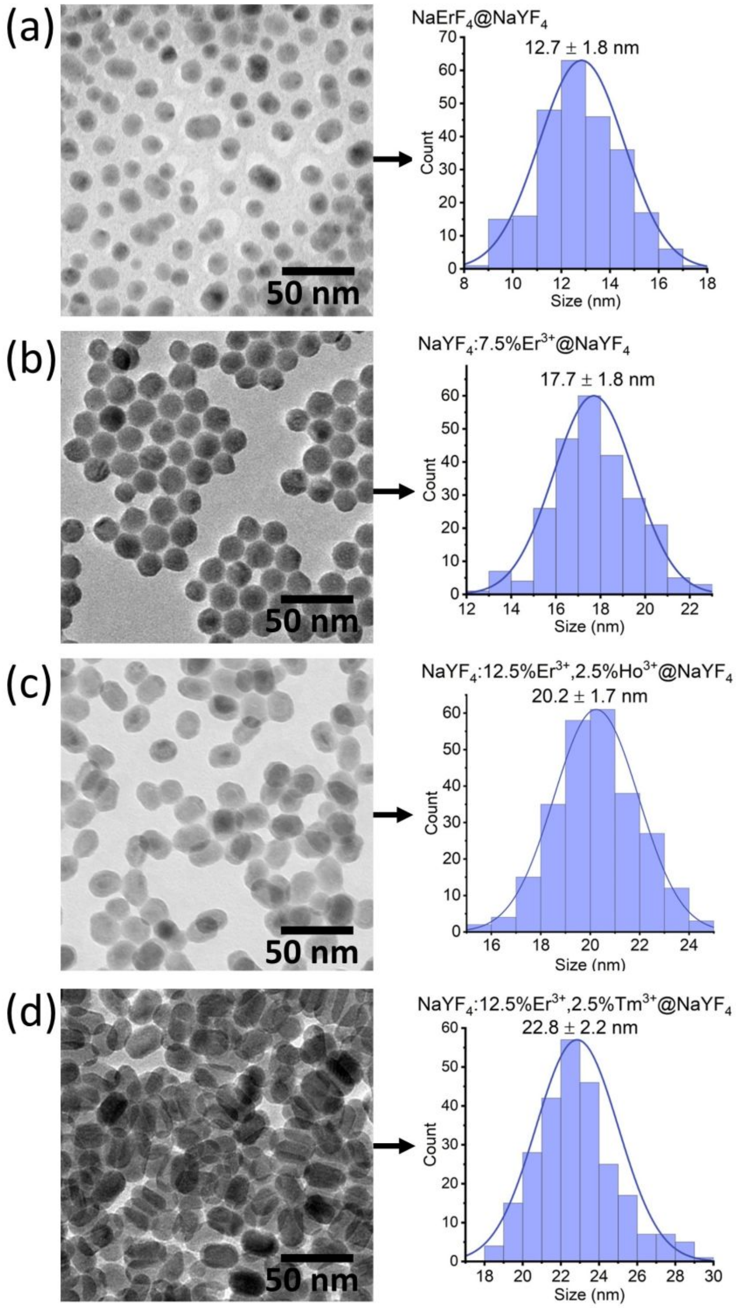

Figure S1. TEM images and NPs' size distributions of OA-capped (a) $NaErF_4$@$NaYF_4$, (b) $NaYF_4$:7.5%Er^{3+}@$NaYF_4$, (c) $NaYF_4$:12.5%Er^{3+},2.5%Ho^{3+}@$NaYF_4$ and (d) $NaYF_4$:12.5%Er^{3+},2.5%Tm^{3+}@$NaYF_4$ NPs.

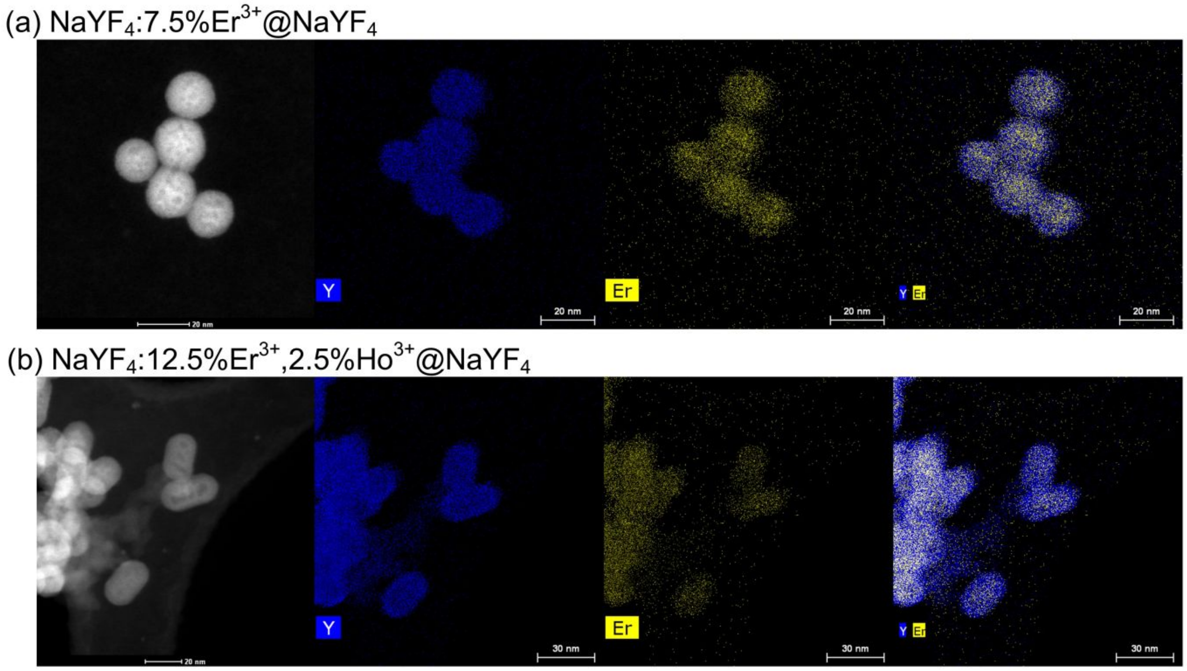

Figure S2. HAADF images (from the left) and Y^{3+}/Er^{3+} ions EDS maps of distribution measured for (a) $NaYF_4$:7.5%Er^{3+}@$NaYF_4$ and (b) $NaYF_4$:12.5%Er^{3+},2.5%Ho^{3+}@$NaYF_4$ NPs.

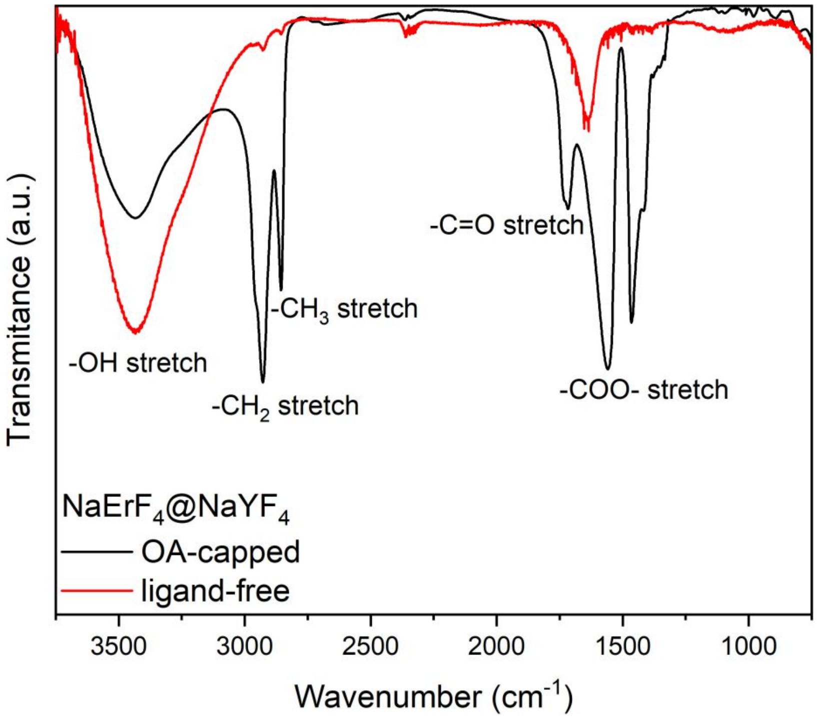

Figure S3. FT-IR spectra of OA-capped and ligand-free $NaErF_4$@$NaYF_4$ NPs confirming ligand removal.

2. Spectroscopic properties

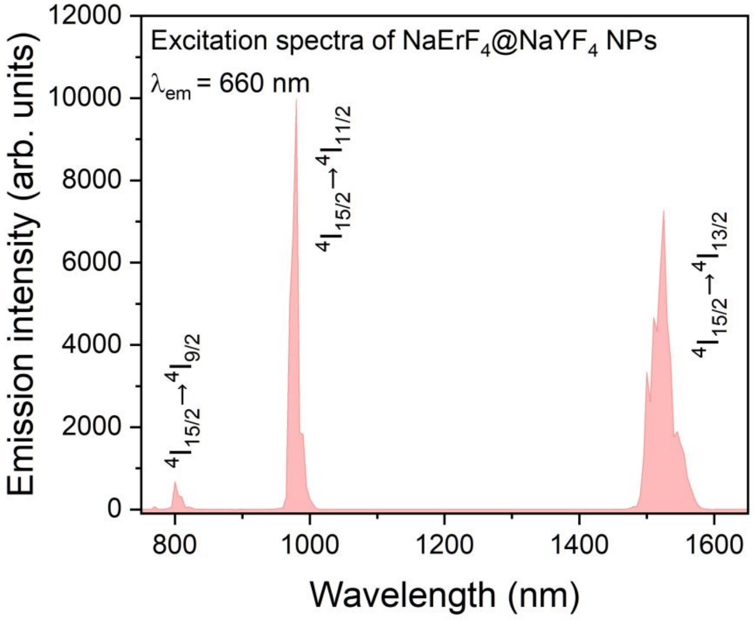

Figure S4. Excitation spectra of $NaErF_4$@$NaYF_4$ NPs measured by observing the emission at 660 nm.

The number of photons needed to fill the emitting levels of Er^{3+}, Ho^{3+} and Tm^{3+} ions used as dopants can be determined using the following equation:[1]

$$I_{UC} \propto P^{n} \tag{S1}$$

Here, I_{UC} denotes the upconversion emission intensity, P stands for the pump laser power density, and n represents the number of photons required. The slope values, obtained from the relationship between upconversion intensity and laser power density plotted on a double-logarithmic scale, serve as n, signifying the photon count. These dependencies, observed for the examined NPs, are illustrated in Figure S5.

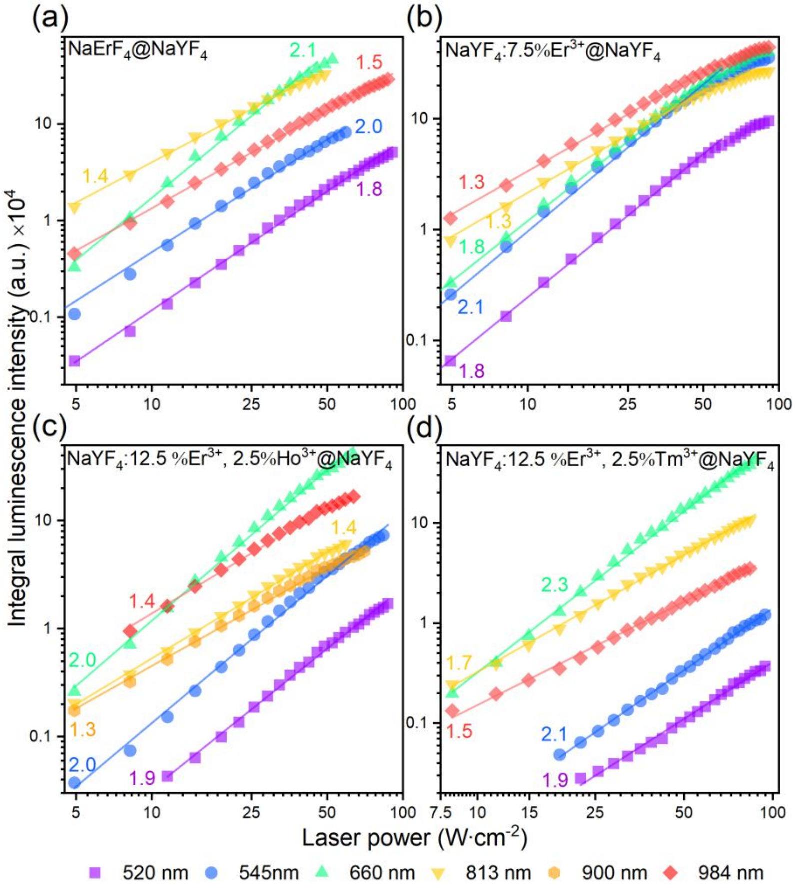

Figure S5. Dependencies of luminescence intensity on 1532 nm laser power density measured for (a) $NaErF_4$@$NaYF_4$, (b) $NaYF_4$:7.5%Er^{3+}@$NaYF_4$, (c) $NaYF_4$:12.5%Er^{3+},2.5%Ho^{3+}@$NaYF_4$ and (d) $NaYF_4$:12.5%Er^{3+},2.5%Tm^{3+}@$NaYF_4$ NPs.

3. Temperature sensing properties

Emission peaks were assessed via integration using Origin Pro 2024 to determine the luminescence intensity ratios (*LIRs*) depicted in Figures S6 and S8. Subsequently, the relationships between *LIRs* and temperature were modelled using two distinct functions. Given the thermal coupling between the $^2H_{11/2}$ and $^4S_{3/2}$ excited states of Er^{3+} ions, the variation of the 520/545 nm *LIR* with temperature was fitted using a Boltzmann-type function:[2]

$$LIR \equiv \frac{I_{520}}{I_{545}} = B \cdot exp\left(-\frac{\Delta E}{k_B T}\right) \tag{S2}$$

where LIR is the luminescence intensity ratio of the emission bands, k_B is the Boltzmann constant, ΔE is the energy separation between the barycenters of the I_{520} and I_{545} bands, T is the absolute temperature, and B is a constant.

The remaining *LIRs* were fitted utilizing a cubic function, which demonstrated superior fitting compared to the Boltzmann-type function, as the excited levels from which the emission was observed were not thermally coupled (Equation S3).[3,4]

$$LIR \equiv \frac{I_1}{I_2} = A + B \times T + C \times T^2 + D \times T^3 \tag{S3}$$

where LIR is the luminescence intensity ratio of the two I_1 and I_2 s mission bands, T is the absolute temperature, and A, B and C are constants. The obtained R^2 correlation coefficients were not lower than 0.99.

The absolute sensitivities, S_a (K^{-1}) were calculated using Equation S4:

$$S_a = \frac{dLIR}{dT} \tag{S4}$$

The relative sensitivities, S_r were calculated using Equation S5. The resultant values are presented in Figures 5 and S8. The S_r value (%·K^{-1}) indicates the extent of change per 1 K of temperature in the measured LIR employed for optical temperature sensing:

$$S_r = \frac{1}{LIR} \cdot \frac{dLIR}{dT} \cdot 100\% = \frac{S_a}{LIR} \cdot 100\% \tag{S5}$$

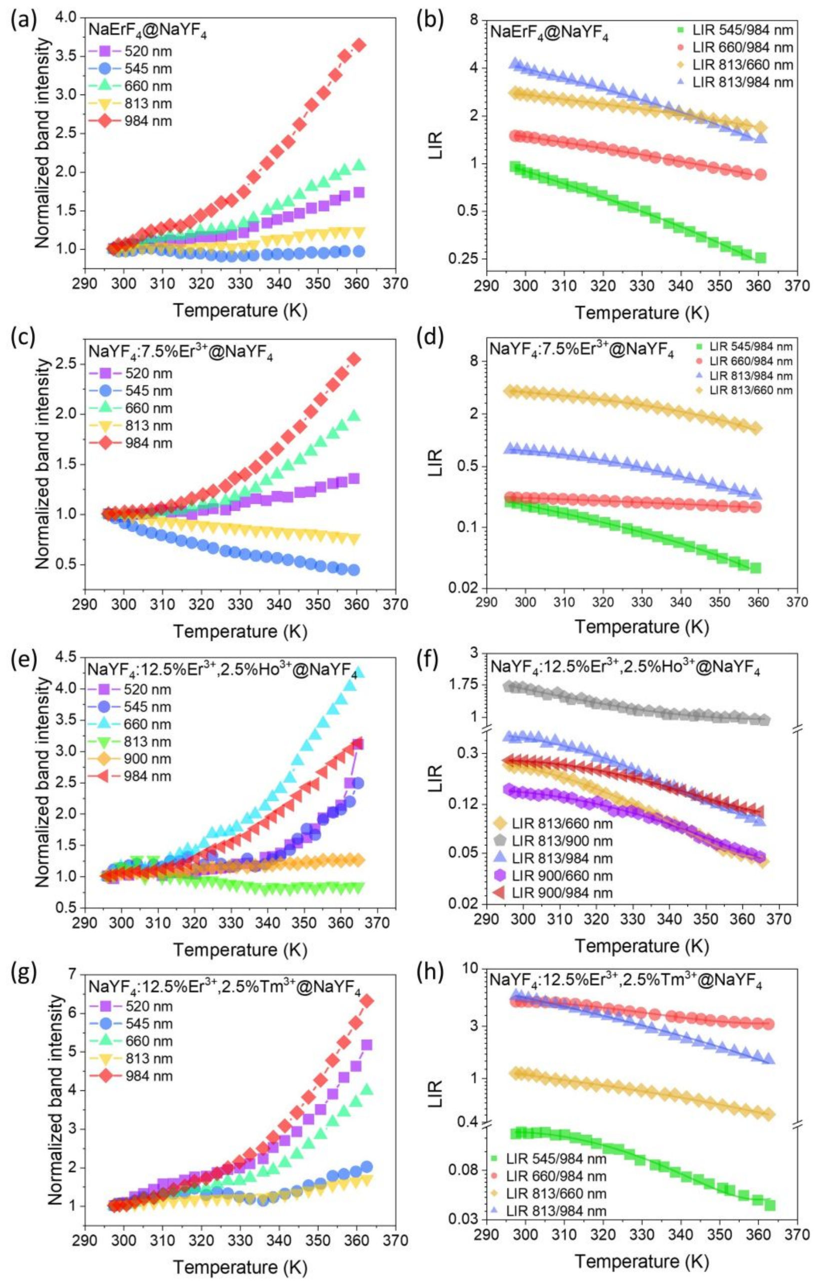

Figure S6. (a, c, e, g) Integrated emission intensity calculated for the measured emission bands under 1532 nm excitation (with a power density of 25 W·cm^{-2}) presented as a function of temperature. The collected dependencies allow for determining which emission bands are the most promising for calculating *LIRs*. (b, d, f, h) *LIRs* calculated based on the collected emission spectra as a function of temperature. The related emission spectra and calculated relative temperature sensitivities are presented in Figure 4 in the main article.

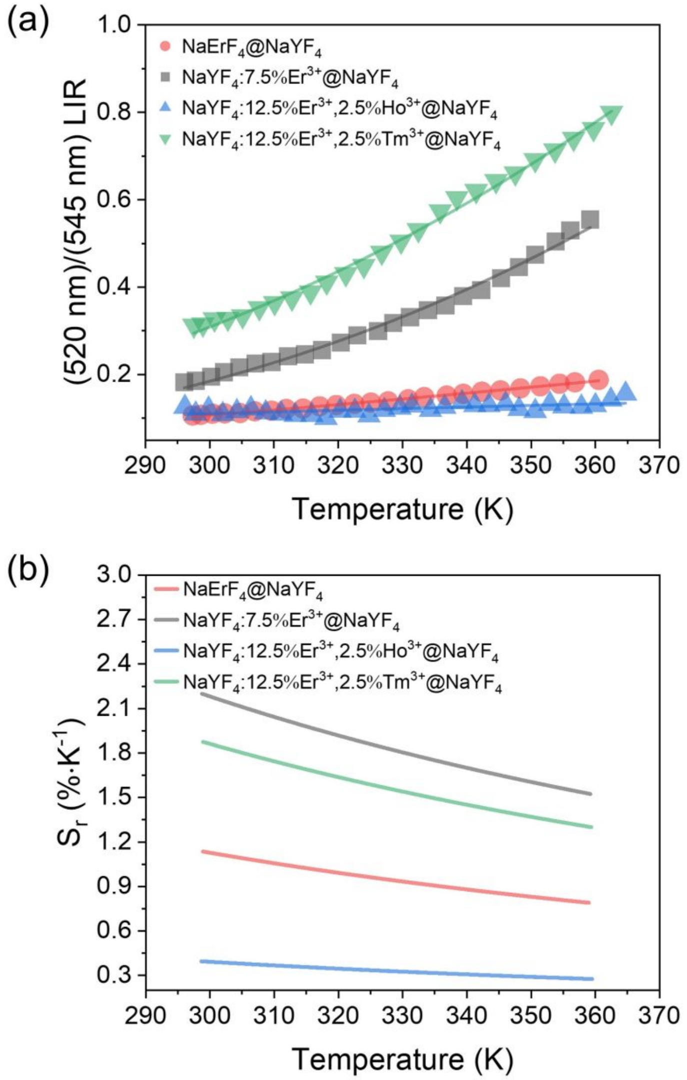

Figure S7. (a) Luminescence intensity ratios calculated based on the emission of Er^{3+} ions related to the thermally coupled levels at 520 and 545 nm under 1532 nm excitation (with a power density of 25 W·cm^{-2}). (b) Relative temperature sensitivities calculated by using the data presented in (a).

Table S1. The calculated absolute and relative temperature sensitivities of NPs for various luminescence intensity ratios are presented. In each cell, the maximum obtained values are listed first, along with the temperature at which this value was achieved, indicated in parentheses. Additionally, the table also shows the sensitivities calculated for human body temperature conditions, *i.e.*, 310 K, to better demonstrate which of the listed nanothermometers are best suited for studies conducted within the range of biological temperatures.

Sample	Calculated absolute (S_a (K^{-1})) and relative (S_r (%·K^{-1})) sensitivities							
	520 nm / 545 nm	545 nm / 984 nm	660 nm / 984 nm	813 nm / 660 nm	813 nm / 900 nm	813 nm / 984 nm	900 nm / 660 nm	900 nm / 984 nm
$NaErF_4$@$NaYF_4$	$S_{r(298\,K)}$ = 1.13<br>$S_{a(298\,K)}$ = 0.0011<br>$S_{r(310\,K)}$ = 1.04<br>$S_{a(310\,K)}$ = 0.0012	$S_{r(359\,K)}$ = 2.81<br>$S_{a(359\,K)}$ = 0.0260<br>$S_{r(310\,K)}$ = 1.91<br>$S_{a(310\,K)}$ = 0.0141	$S_{r(298\,K)}$ = 1.12<br>$S_{a(298\,K)}$ = 0.0094<br>$S_{r(310\,K)}$ = 0.85<br>$S_{a(310\,K)}$ = 0.0115	$S_{r(359\,K)}$ = 0.98<br>$S_{a(359\,K)}$ = 0.0167<br>$S_{r(310\,K)}$ = 0.67<br>$S_{a(310\,K)}$ = 0.0170	-	$S_{r(max)}$ = 2.22 $^{(359\,K)}$<br>$S_{a(max)}$ = 0.0315<br>$S_{r(310\,K)}$ = 1.46<br>$S_{a(310\,K)}$ = 0.0501	-	-
$NaYF_4$:7.5%Er^{3+} @$NaYF_4$	$S_{r(298\,K)}$ = 2.19<br>$S_{a(298\,K)}$ = 0.0038<br>$S_{r(310\,K)}$ = 2.07<br>$S_{a(310\,K)}$ = 0.0046	$S_{r(359\,K)}$ = 4.55<br>$S_{a(359\,K)}$ = 0.0084<br>$S_{r(310\,K)}$ = 2.26<br>$S_{a(310\,K)}$ = 0.0032	$S_{r(359\,K)}$ = 0.48<br>$S_{a(359\,K)}$ = 0.0008<br>$S_{r(310\,K)}$ = 0.38<br>$S_{a(310\,K)}$ = 0.0008	$S_{r(359\,K)}$ = 2.51<br>$S_{a(359\,K)}$ = 0.0327<br>$S_{r(310\,K)}$ = 1.01<br>$S_{a(310\,K)}$ = 0.0325	-	$S_{r(349\,K)}$ = 2.68<br>$S_{a(349\,K)}$ = 0.0081<br>$S_{r(310\,K)}$ = 1.28<br>$S_{a(310\,K)}$ = 0.0086	-	-
$NaYF_4$:12.5%Er^{3+},2.5%Ho^{3+} @$NaYF_4$	$S_{r(298\,K)}$ = 0.39<br>$S_{a(298\,K)}$ = 0.0004<br>$S_{r(310\,K)}$ = 0.35<br>$S_{a(310\,K)}$ = 0.0004	-	-	$S_{r(343\,K)}$ = 3.48<br>$S_{a(343\,K)}$ = 0.0027<br>$S_{r(310\,K)}$ = 1.93<br>$S_{a(310\,K)}$ = 0.0038	$S_{r(296\,K)}$ = 1.33<br>$S_{a(296\,K)}$ = 0.0228<br>$S_{r(310\,K)}$ = 1.22<br>$S_{a(310\,K)}$ = 0.0175	$S_{r(343K)}$ = 3.17<br>$S_{a(343K)}$ = 0.0049<br>$S_{r(310\,K)}$ = 1.56<br>$S_{a(310\,K)}$ = 0.0054	$S_{r(355\,K)}$ = 2.56<br>$S_{a(355\,K)}$ = 0.0015<br>$S_{r(310\,K)}$ = 1.05<br>$S_{a(310\,K)}$ = 0.0014	$S_{r(359\,K)}$ = 1.94<br>$S_{a(359\,K)}$ = 0.0022<br>$S_{r(310\,K)}$ = 0.80<br>$S_{a(310\,K)}$ = 0.0020
$NaYF_4$:12.5%Er^{3+},2.5%Tm^{3+} @$NaYF_4$	$S_{r(298\,K)}$ = 1.87<br>$S_{a(298\,K)}$ = 0.0056<br>$S_{r(310\,K)}$ = 1.73<br>$S_{a(310\,K)}$ = 0.0064	$S_{r(343\,K)}$ = 3.46<br>$S_{a(343\,K)}$ = 0.0023<br>$S_{r(310\,K)}$ = 1.26<br>$S_{a(310\,K)}$ = 0.0019	$S_{r(333\,K)}$ = 1.05<br>$S_{a(333\,K)}$ = 0.0406<br>$S_{r(310\,K)}$ = 0.67<br>$S_{a(310\,K)}$ = 0.0320	$S_{r(360\,K)}$ = 1.74<br>$S_{a(360\,K)}$ = 0.0085<br>$S_{r(310\,K)}$ = 1.09<br>$S_{a(310\,K)}$ = 0.0105	-	$S_{r(360\,K)}$ = 2.85<br>$S_{a(360\,K)}$ = 0.0418<br>$S_{r(310\,K)}$ = 1.87<br>$S_{a(310\,K)}$ = 0.0845	-	-

To estimate the uncertainty of the temperature measurement, we performed a measurement of 100 emission spectra at room temperature. The methodology for measuring uncertainty involved calculating the *LIR* from each of the collected spectra and then using the functions obtained by fitting the relationship between *LIR* and temperature, as shown by the solid lines in Figs. S6b, d, f, and h, as well as Fig. 7a. The temperature corresponding to the measured *LIR* was then determined. From the 100 temperature values obtained in this way, the standard deviation from room temperature was calculated, which corresponds to the uncertainty of the temperature measurement.

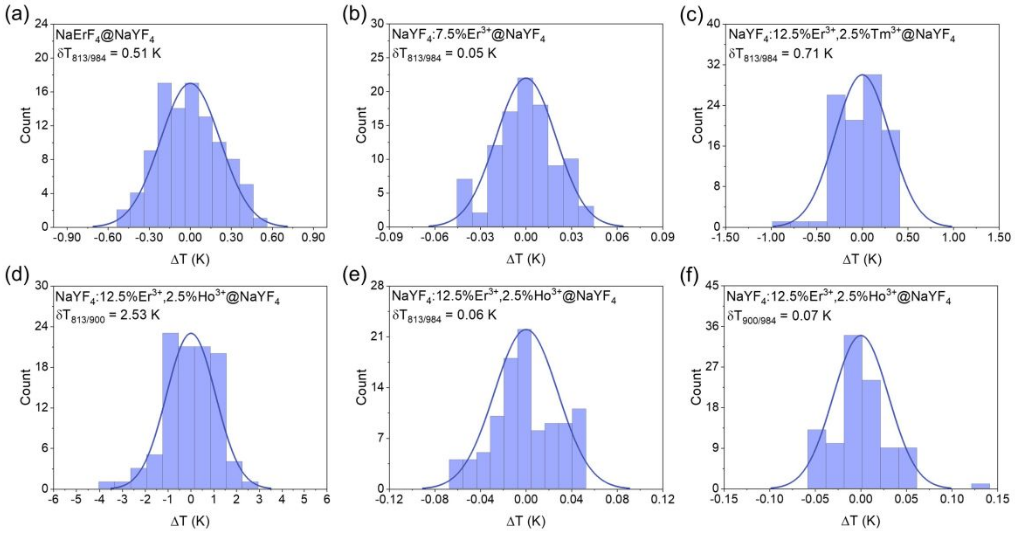

Figure S8. Temperature uncertainties calculated based on 100 measurements of emission spectra for (a) $NaErF_4$@$NaYF_4$, (b) $NaYF_4$:7.5%Er^{3+}@$NaYF_4$, (c) $NaYF_4$:12.5%Er^{3+},2.5%Tm^{3+} and (d-f) $NaYF_4$:12.5%Er^{3+},2.5%Ho^{3+}@$NaYF_4$ at room temperature (296 K).

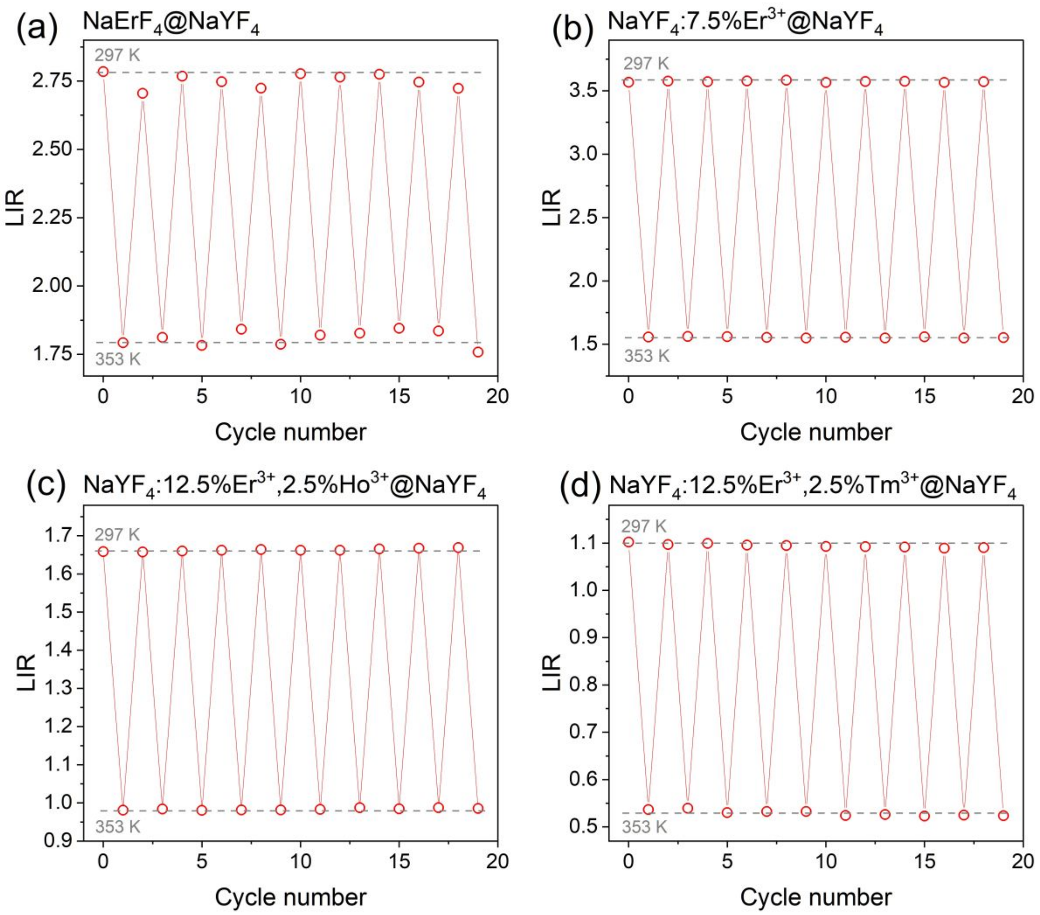

Figure S9. The changes in LIRs between the 813 nm and 660 nm bands from 297 K to 353 K as a function of cycle number for (a) $NaErF_4$@$NaYF_4$, (b) $NaYF_4$:7.5%Er^{3+}@$NaYF_4$, (c) $NaYF_4$:12.5%Er^{3+},2.5%Ho^{3+}@$NaYF_4$and (d) $NaYF_4$:12.5%Er^{3+},2.5%Tm^{3+}, under excitation with a 1532 nm laser (power density: 25 W·cm^{-2}).

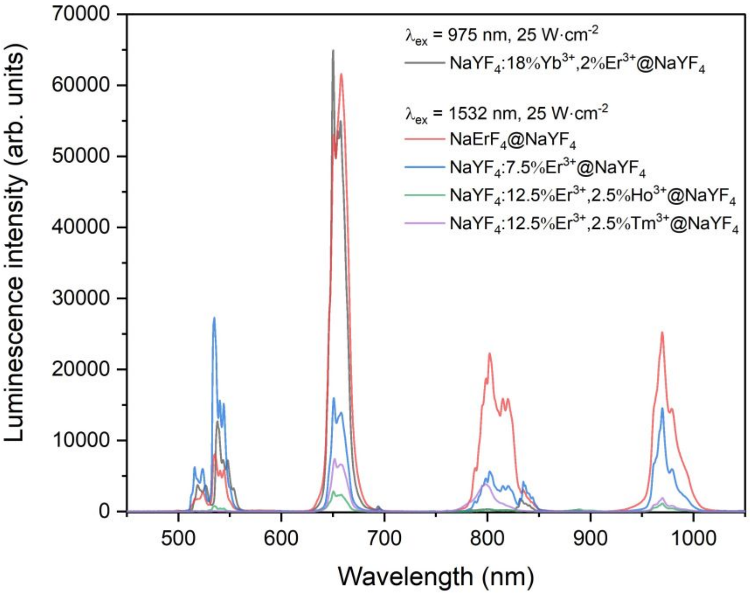

Figure S10. Comparison of the luminescence of the obtained nanoparticles to the emission of $NaYF_4$:18%Yb^{3+},2%Er^{3+}@$NaYF_4$ nanoparticles with a known quantum emission efficiency of 0.67 ± 0.11% under the influence of a 975 nm laser.[5] The above spectra were measured while maintaining the same excitation power density for both excitation lasers.

To estimate the quantum yield of upconversion (UCQY) for NPs under the influence of a 1532 nm laser, we first measured the emission of NPs with a known quantum yield. Then, maintaining the same power density of the excitation laser, we measured the emission of NPs sensitized with Er^{3+} ions under the influence of the 1532 nm laser. The emission intensity of the best-emitting NPs, *i.e.*, $NaErF_4$@$NaYF_4$, is comparable to $NaYF_4$:18%Yb^{3+},2%Er^{3+}@$NaYF_4$ NPs. However, the emission of the former is significantly more intense in the 750–1050 nm range, suggesting that the UCQY could be even higher than the reported 0.67 ± 0.11% for NPs excited by a 975 nm laser. To estimate the UCQY value, one can assume that the UCQY is proportional to the area under the recorded emission bands. Calculations are presented in Table S2.

Table S2. The estimated UCQY of the obtained NPs calculated based on a comparison of their emission with that of NPs with a known UCQY under the same power density of the excitation laser.

Sample	Integrated emission intensity (arb. units)	UCQY
$NaYF_4$:18%Yb^{3+},2%Er^{3+}@$NaYF_4$	$1.27\cdot10^6$	0.67 ± 0.11 %[1]
$NaErF_4$@$NaYF_4$	$2.57\cdot10^6$	~1.36
$NaYF_4$:7.5%Er^{3+}@$NaYF_4$	$1.04\cdot10^6$	~0.55
$NaYF_4$:12.5%Er^{3+},2.5%Ho^{3+}@$NaYF_4$	$9.36\cdot10^4$	~0.05
$NaYF_4$:12.5%Er^{3+},2.5%Tm^{3+}@$NaYF_4$	$2.64\cdot10^5$	~0.07

4. References

(1) Pollnau, M.; Gamelin, D.; Lüthi, S.; Güdel, H.; Hehlen, M. Power Dependence of Upconversion Luminescence in Lanthanide and Transition-Metal-Ion Systems. *Phys. Rev. B* **2000**, *61* (5), 3337–3346. https://doi.org/10.1103/PhysRevB.61.3337.

(2) Quintanilla, M.; Henriksen-Lacey, M.; Renero-Lecuna, C.; Liz-Marzán, L. M. Challenges for Optical Nanothermometry in Biological Environments. *Chem. Soc. Rev.* **2022**, *51* (11), 4223–4242. https://doi.org/10.1039/D2CS00069E.

(3) Nexha, A.; Pujol, M. C.; Carvajal, J. J.; Díaz, F.; Aguiló, M. Luminescence Nanothermometry via White Light Emission in Ho^{3+}, Tm^{3+}:Y_2O_3 Colloidal Nanocrystals. *J. Lumin.* **2022**, *247*, 118854. https://doi.org/10.1016/j.jlumin.2022.118854.

(4) Lu, H.; Hao, H.; Gao, Y.; Li, D.; Shi, G.; Song, Y.; Wang, Y.; Zhang, X. Optical Sensing of Temperature Based on Non-Thermally Coupled Levels and Upconverted White Light Emission of a $Gd_2(WO_4)_3$ Phosphor Co-Doped with in Ho(III), Tm(III), and Yb(III). *Microchim. Acta* **2017**, *184* (2), 641–646. https://doi.org/10.1007/s00604-016-2070-6.

(5) Jurga, N.; Przybylska, D.; Kamiński, P.; Tymiński, A.; Grześkowiak, B. F.; Grzyb, T. Influence of the Synthesis Route on the Spectroscopic, Cytotoxic, and Temperature-Sensing Properties of Oleate-Capped and Ligand-Free Core/Shell Nanoparticles. *J. Colloid Interface Sci.* **2022**, *606*, 1421–1434. https://doi.org/10.1016/j.jcis.2021.08.093.
